# Drug-susceptibility Patterns of *Mycobacterium tuberculosis* in Mpumalanga Province, South Africa: Possible Guiding Design of Retreatment Regimen

**DOI:** 10.3329/jhpn.v28i1.4518

**Published:** 2010-02

**Authors:** E. Green, C.L. Obi, M. Nchabeleng, B.E. de Villiers, P.P. Sein, T. Letsoalo, A.A. Hoosen, P.O. Bessong, R.N. Ndip

**Affiliations:** ^1^ AIDS Virus Research Laboratory, Department of Microbiology, University of Venda, South Africa; ^2^ Department of Biochemistry and Microbiology, University of Fort Hare, Private Bag X1314, Alice 5700, South Africa; ^3^ Research and Academic Directorate, Walter Sisulu University, Mthatha, South Africa; ^4^ Deparment of Medical Microbiology, University of Pretoria, South Africa; (*Deceased); ^5^ Department of Biochemistry and Microbiology, University of Buea, Cameroon

**Keywords:** Drug resistance, Microbial, *Mycobacterium tuberculosis*, Risk factors, Tuberculosis, South Africa

## Abstract

Multidrug-resistant tuberculosis (MDR-TB) has been a cause of concern in both developed and developing countries. The prevalence of drug resistance in *Mycobacterium tuberculosis* (MTB) isolates (n=692) from Mpumalanga province was assessed. In total, 692 (64%) MTB strains from cases with pulmonary TB were tested for susceptibility against rifampicin, isoniazid, ethambutol, and streptomycin using the MGIT 960 instrument. Two hundred and nine (30.2%) strains were resistant to one or more drugs. Resistance to one drug ranged from 1.4% for ethambutol to 17.7% for rifampicin. The prevalence of MDR-TB ranged from 6.7% for three drugs to 34% for four drugs, with significant predictors being patients’ age-groups of 25–54 years (p=0.0012) and >55 years (p=0.007). The result showed a high level (58.4%) of MDR-TB from cases in Mpumalanga province. To achieve a higher cure rate in this province, drug-susceptibility tests must be done for every case.

## INTRODUCTION

Tuberculosis (TB) remains a deadly infectious disease, affecting millions of people worldwide. Although it is a global epidemic, TB predominantly affects populations in resource-poor countries, where 98% of all deaths due to TB occur (1). Worldwide, two billion people are latently infected with *Mycobacterium tuberculosis* (MTB). In 2005, 8.9 million new cases of TB were recorded, and 1.8 million deaths were attributed to the disease (2, 3). According to the World Health Organization (WHO), there were an estimated 500,000 cases of MDR-TB in 2007 (4). It is estimated that the actual number of new cases is three times the number of cases reported (5). One-third of 40 million people currently infected with human immunodeficiency virus (HIV) are co-infected with TB (6, 7). The changing patterns of global health events over the last two decades, particularly the spread of HIV and drug-resistant TB, have aided in posing serious healthcare problems, thus necessitating the advancement of alternative strategies for effectively combating these re-emerging problems. Strains of MTB resistant to anti-mycobacterial agents, including isoniazid (INH) and rifampicin (RIF), with or without resistance to other first-line drugs, have been reported worldwide (8). Recently-described extensively drug-resistant TB (XDR-TB) has been reported in South Africa (9–11).

The WHO recommends standardized TB treatment regimens based on short-course chemotherapy. The anti-TB drug regimen recommended for the treatment of new cases consists of two months of INH, RIF, pyrazinamide (PZA), and ethambutol (EMB) administration, followed by four months of INH and RIF (12). This treatment is usually effective against wild MTB strains that have never been exposed to anti-TB drugs for more than 30 days (13). The most common indicator of drug-resistant (DR) and multidrug-resistant tuberculosis (MDR-TB) is a history of incorrect treatment of TB (14, 15).

Continuous surveillance of primary (infection with a drug-resistant strain) and secondary drug resistance (selection and growth of resistant population) patterns of MTB is important in assessing the efficacy of chemotherapy regimens used in the past years and in detecting the problems in past treatments (16). Attention has focused on assessing the global burden of MDR-TB and predicting the future threat of this pathogen. Some investigators predict a global pandemic of MDR-TB whereas others see it as a local problem that can be managed by properly implementing the currently-recommended strategies (14). Despite this attention, there is little consensus on either the magnitude of the problem or its future trend.

There is a paucity of information in primary drug resistance among MTB strains circulating in Mpumalanga province, South Africa. The present study was, therefore, undertaken to measure the prevalence of DR-TB, including MDR-TB, in Mpumalanga province. These results will contribute to guiding the design of retreatment regimen for DR-TB and MDR-TB in the province.

## MATERIALS AND METHODS

### Study population

One thousand and eighty-four sputum samples were collected from 18 districts in Mpumalanga province, South Africa. The inclusion criteria for collecting sputum specimens from patients were according to the Department of Health guidelines, which include clinically-suspected TB, treatment failure, and retreatment among patients. Patients, aged 15–71, years presenting to different clinics and hospitals and outpatient facilities in Mpumalanga during January 2004–December 2006 were eligible for the study. Informed consent was obtained from all patients before enrollment. The Research and Ethics Committee of the University of Venda and the Department of Health and Welfare, Mpumalanga province, approved the study.

### Collection of data and definitions

Clinical, demographic, and epidemiologic data were collected by interviewing patients. Demographic data included age of patient, gender, region of residence, and history of treatment. The study focused primarily on patients who had positive cultures for TB. Multidrug resistance (MDR) was defined as resistance to at least both INH and RIF (17, 18) with or without resistance to other drugs. Monoresistance was defined as resistance exclusively to one of the four first-line anti-TB drugs tested (16). Polyresistance was defined as resistance to two or more of the four first-line anti-TB drugs but not both INH and RIF.

### Laboratory methods

Three sputum samples for acid-fast bacillus (AFB) smear microscopy and culture were obtained from each patient enrolled in the study. Specimens were obtained at each district and then transported to TB Laboratory of Dr George Mukhari Hospital in Pretoria where AFB cultures were performed in the BacT/Alert 3D system (BA, bioMérieux, Durham, USA). Dr George Mukhari Hospital is a 1,200-bed tertiary teaching and referral hospital attached to the Medunsa Campus of the University of Limpopo. Sputum specimens are routinely sent to Microbiology Laboratory of Dr George Mukhari Hospital for analysis of TB. Positive cultures were stained by the Ziehl-Neelsen technique. If more than one culture-positive specimen per patient were processed, only one positive result was registered for the patient; similarly, if several negative cultures per patient were encountered, without a positive finding, only one negative result was recorded. Confirmation of MTB was done using the AccuProbe DNA hybridization assay (GenProbe) following the instructions of the manufacturer. All positive cultures were advanced for susceptibility testing.

### Susceptibility tests

Susceptibility to all culture-positive MTB strains was done for the first-line anti-TB drugs, including INH, RMP, and EMB and one second-line drug SM using the MGIT 960 instrument (Becton Dickinson Microbiology Systems, Sparks, MD, USA) (19). The concentrations of drugs were 0.2, 40.0, 2.0, and 4.0 μg/mL for INH, RMP, EMB, and SM respectively. Internal controls were used for ensuring the quality of drug-susceptibility test.

A standard questionnaire and the register were used for determining the history of previous anti-TB drug therapy, and the patients were categorized as having either initial or secondary resistance, according to the standard definitions (18). Resistance to INH and RIF with or without resistance to other anti-TB drugs was defined as multidrug resistance.

### Statistical analysis

All statistical analyses were performed using the SPSS software (version 16.0) and the Epi Info software (version 3.4.1). Risk factors for having non-multidrug resistance and multidrug resistance among culture-confirmed TB cases were assessed. Univariate analysis was performed to determine unadjusted association of TB drug resistance with demographic characteristics of patients. The prevalence ratios (PRs) with 95% confidence interval (CI) were calculated using StatCalc on Epi Info. A p value of ≤0.05 was considered statistically significant.

## RESULTS

### Characteristics of patients

Of the 1,084 sputum specimens received, 692 (63.8%) were positive for MTB culture. Drug-susceptibility test and demographic data of the 692 strains were analyzed (Table 1). The results showed that 209 (30.2%) strains were resistant to at least one or more drugs. Of the 209 resistant strains, 100 (47.8%) were isolated from female patients with a mean age of 35.08 years while 109 (52.2%) were from males with a mean age of 36.70 years. Seventy-two (34.4%) patients were hospitalized, 137 (65.5%) were outpatients, and eight (3.8%) outpatients were prisoners. Having a positive culture for *M. tuberculosis* was associated with male gender (Table 1).

**Table 1.** Univariate analysis of association of having resistance to at least one first-line anti-tuberculosis drug with demographic characteristics of patients

Variable	Culture-positive (n=692)		Resistance (n=209)		Susceptible (n=483)		PR (95% CI)	p value
	No.	%	No.	%	No.	%		
Age-group (years)								
15–24	26	4	18	8.6	8	1.7	Ref*	1
25–34	72	10	68	33	4	0.8	1.36 (1.05–1.77)	0.002
35–44	206	30	20	9.6	186	38.5	0.44 (0.09–0.23)	0.000
45–54	89	13	32	15.3	57	11.8	0.52 (0.36–0.76)	0.005
55–64	215	31	35	16.7	180	37	0.24 (0.16–0.35)	0.000
>65	84	12	36	17.2	48	9.9	0.62 (0.43–0.88)	0.003
Gender								
Male	413	60	109	52.2	304	63	0.74 (0.59–0.92)	0.01
Female	275	40	100	47.8	179	37	Ref*	1

*Values were used as a reference to compare;

CI=Confidence interval;

PR=Prevalence ratio;

Ref=References

### Risk of drug-resistant tuberculosis

The prevalence of different patterns of resistance is shown in Table 2. Overall, 209 strains were resistant to one or more drugs. The proportion of new TB cases with resistance to a single drug ranged from 1.4% for EMB to 17.7% for INH, with a median value of 4.5%. MDR-TB showed a prevalence range of 6.7% for INH+RIF+EMB/SM to 34% for INH+RIF+SM+EMB, with a median of 14.6%. The prevalence of polyresistance to anti-TB drugs tested ranged from 0.5% for RIF+SM to 6.7% for INH+SM+EMB (a median of 0.7%) (Table 2). The overall resistance to drugs tested ranged from 28% for INH to 88% for EMB, with a median of 61%.

**Table 2.** Prevalence of first-line anti-tuberculosis resistance of 209 *Mycobacterium tuberculosis* strains from Mpumalanga province

Resistance profile	All cases	
	No.	%
Total resistance	209	100
Monoresistance	59	28.2
INH only	37	17.7
RIF only	13	6.1
SM only	6	2.9
EMB only	3	1.4
MDR	122	58.4
INH+RIF	23	11
INH+RIF+EMB	14	6.7
INH+RIF+SM	14	6.7
INH+RIF+SM+EMB	71	34
Polyresistance*	28	13.4
INH+SM+EMB	14	6.7
INH+SM	11	5.3
RIF+SM	1	0.5
EMB+SM	2	1
Overall resistant to		
INH drug	204	97.6
RIF drugs	136	65.1
SM drugs	119	57
EMB drugs	104	49.8

*Resistant to two or more drugs but not both isoniazid and rifampicin;

EMB=Ethambutol;

INH=Isoniazid;

MDRTB=Multi-drug-resistant tuberculosis (resistant to at least both isoniazid and rifampicin with or without resistance to other drugs);

RIF=Rifampicin;

SM=Streptomycin

There was no significant difference between the MDR-TB cases and the non-MDR-TB cases (49%) (p=0.6) in male and female cases respectively (Table 3). A slightly higher prevalence (54%) of MDR-TB cases was observed in males than in females (46%). However, for the non-MDR-TB cases, a slightly higher prevalence (51%) was observed in females than in males (49%) (Table 3). The significant predictor of MDR-TB in univariate analysis included patients aged 35–54 years (prevalence ratio [PR]=1.84, 95% CI 1.37–2.46, p value for trends=0.0012) and patients aged >54 years (PR=1.55, 95% CI 1.14–2.09, p value for trends=0.007) (Table 3).

**Table 3.** Univariate analysis of association of having MDR-TB with demographic characteristics of patients

Variable	Total resistance (n=209)		MDR-TB cases (n=122)		Non-MDR-TB cases (n=87)		PR (95% CI)	p value
	No.	%	No.	%	No.	%		
Age-group (years)								
15–34	86	41	36	29.5	50	57	1 0.70–1.42	1.00
35–54	52	25	40	36	12	13	1.84 (1.37–2.46)	0.0012
55–75	71	34	46	41	25	29	1.55 (1.14–2.09)	0.007
Gender								
Male	109	52	66	54	43	49	1.08 (0.86–1.36)	0.6
Female	100	48	56	46	44	51	1 0.78–1.28	1.00

CI=Confidence interval;

MDR=Multidrug-resistant;

PR=Prevalence ratio;

TB=Tuberculosis

## DISCUSSION

South Africa is one of the 22 high-burden countries that contribute approximately 80% of the total global burden of all TB cases (7). The country has the seventh highest incidence of TB in the world. In South Africa, the incidence of TB has increased, in parallel to the increase in the estimated prevalence of HIV among adults during the past 10 years (11), resulting in the increasing recognition of the problems posed to public health by TB. The risk of DR-TB transmission can be reduced by efficient diagnosis and timely treatment of DR-TB patients (10). Thus, the DR status of a patient needs to be confirmed before treatment. Better access to drug-susceptibility test results at the time of diagnosis of TB would facilitate appropriate selection of treatment regimens, thereby minimizing the development of DR strains.

The main focus in the twentieth century was to cure TB and minimize its transmission to other persons (20). The WHO developed treatment regimen categorized according to different clinical presentations. Management of new patients suspected of having contracted DR strains of *M. tuberculosis* is essentially similar to the treatment of other new patients. Standardized treatment regimen (STR) in South Africa is given to all patients in a population based on the history of drug used in the country and a representative drug-susceptibility testing.

This study indicated monoresistance to all drugs tested. Overall, we found that resistance to INH (17.7%) and RIF (6.1%) was more prevalent than in other agents. The high resistance of MTB to INH and RIF—the two main drugs in TB treatment—is an alarming sign. In other studies, SM and INH have been reported as more common compared to the other first-line drugs (21, 22). Other patterns of anti-TB resistance also existed. In our study, resistance to SM and EMB was less prevalent. Resistance to SM may have been selected following previous and regular administration in the treatment of other bacterial diseases. This finding may indicate a risk *vis-a-vis* the use of WHO category II (CAT II) regimen for retreatment cases in Mpumalanga. About 28.2% and 13.4% of the strains were resistant to mono-drug and poly-drugs (non-MDR) respectively. Thus, approximately 41.6% of the patients in this study needed to have their treatment changed, as the use of standard therapy in mono-DR-TB and poly-DR-TB has a high risk of failure and can lead to the development of MDR-TB in these patients (23).

More than half (58.4%) of our study patients were infected with MDR strains. This is different from what was obtained in Cuba during 1999–2000 where 0.5% of MDR-TB was obtained (24). The relationship between history of receiving anti-TB treatment and drug resistance has been clearly described (25). The prevalence of MDR-TB among patients diagnosed with TB was 16.3% in mid-1990 (26). In a study conducted in Mpumalanga during 2004–2006 showed a marked decrease in the prevalence of MDR-TB, from 1.92% to 0.7% (27). The difference could be explained by the criteria used for the inclusion of patients in this study. Our sample was a convenience sample (patients were requested to give the sputum specimen when they were at the clinics or hospitals) while the National Department of Health did a population-based study. The high number of isolated MDR-TB isolates in our study is of concern in Mpumalanga where the TB-control programme has not been functioning optimally. In general, a high rate of MDR-TB among patients is suggestive of poor management of past and existing disease (27). MDR-TB is associated with higher rates of failure and death, is more difficult and expensive to treat than drug-susceptible TB (7), and has an increased risk of adverse drug-effects for patients (28).

Knowledge-base for future intervention can be provided by identifying risk factors for infection, disease, and MDR-TB (29, 30). In our study, the strongest risk factor of having drug resistance was the age-group of 35–44 years and 55–60 years (p< 0.001). In line with other reports (31, 32), the risk of MDR-TB was observed in the age-group of 35–54 years (p=0.0012 at 95% CI) and >54 years (p=0.007 at CI 95%). These results could be a reflection of the 2005 situation when directly-observed therapy, short-course strategy (DOTS) was interrupted in Mpumalanga (26). Elderly persons had been exposed to the organisms in the past, and during the process of treatment, resistance was acquired while young patients are more likely to have acquired the bacilli more recently when they were more likely to be resistant.

In our study, TB, including MDR-TB, was more common among males than among females. Other researchers also observed this trend. In a systematic review, MDR-TB cases were more likely to be associated with males in Western Europe while, in Eastern Europe, the male gender was not associated with MDR-TB (31, 33). It was assumed that the male gender could modify the association between treatment and MDR-TB since men are believed to be less adherent to treatment than women are.

The spread of DR-TB in Mpumalanga could seriously hamper efforts to control TB. Limited resources and laboratory capacity are a few limitations in the proper control of TB. Increasing the laboratory capacity to deal with DR-TB as recommended by the Global Plan to Stop TB and better referral of specimens from patients living in remote areas to regional or national reference laboratories should be encouraged to ensure success of the TB-control programme and control transmission of DR strains.

The study encountered some limitations. One of these was the potential misclassification of new and retreatment cases; some cases registered as new may actually have had treatment for TB in the past. Classification was based on the patient's history of prior treatment for TB and review of medical records (which were available for all but four patients enrolled). Due to this reason, categorizing the cases as new or retreatment was omitted. Our study, carried out at selected sites in Mpumalanga, was not population-based, which can introduce bias. The high rate of missing data (demographic profile) among patients with a negative AFB culture was because the study focused primarily on patients with a positive culture. However, our study provides important continual data on drug resistance in Mpumalanga.

Although the sample size is small, the results suggest that multidrug resistance is likely to pose a significant threat to health and impede the success of TB management in Mpumalanga, if adequate control measures are not implemented. Monitoring the trend of resistance should remain a priority.

## ACKNOWLEDGEMENTS

The study was supported by the National Research Foundation, South Africa. The authors are thankful to the laboratory management for allowing them to work in their departmental laboratory at the University of Limpopo, Medunsa Campus. They thank the staff of the TB section, Microbiological Pathology Laboratory, NHLS, Dr George Mukhari Tertiary Laboratory Complex, Pretoria, for technical assistance. The authors dedicate this work to their colleague Dr. P.P. Sein who passed away recently.
